# Increased Depression and the Worsening of Depressive Symptoms Associated with Physical Inactivity during the COVID-19 Pandemic: A Two-Phase Cross-Sectional Study

**DOI:** 10.3390/bs13040340

**Published:** 2023-04-19

**Authors:** Luana Lemos Leão, Weslley Gomes de Araújo Valadares, Nayra Suze Souza e Silva, Stênio Fernando Pimentel Duarte, Alfredo Maurício Batista de Paula, Desirée Sant´Ana Haikal, Sérgio Henrique Sousa Santos, Jeane Soares, Joelton Cruz, Jordana Santos, Gustavo Leal Teixeira, Renato Sobral Monteiro-Junior

**Affiliations:** 1 Graduate Program in Health Sciences, Centre of Biological Sciences and Health, State University of Montes Claros, Montes Claros 39401-089, MG, Brazil; 2 Instituto de Pesquisa e Extensão em Saúde-INPES, Vitória da Conquista 45020-750, BA, Brazil; 3 Food Engineering Department, Instituto de Ciências Agrárias, Universidade Federal de Minas Gerais, Montes Claros 39404-547, MG, Brazil; 4 Department of Psychology, UniFG University Center, Guanambi 46430-000, BA, Brazil; 5 Language Department, Instituto de Ciências Agrárias, Universidade Federal de Minas Gerais, Montes Claros 39404-547, MG, Brazil; 6 Department of Neurology, School of Medicine, Universidade Federal Fluminense, Niterói 24220-000, RJ, Brazil

**Keywords:** sedentary behavior, depressive disorder, pandemics, mental health

## Abstract

The COVID-19 pandemic has increased the incidence of depression and other mental disorders in the general population, influenced by various individual and contextual factors. Physical activity (PA) interventions offer a promising approach to mitigating the negative mental health effects of the pandemic. This study aims to analyze the association between PA and depressive symptoms. A total of 785 individuals aged 37.4 ± 13.2 years (72.5% female) were evaluated at two different time points: the first between 2018 and 2019, and the second during the COVID-19 pandemic in 2020. Depressive symptoms, demographic, and socioeconomic data were assessed using the Beck Depression Inventory to estimate depressive symptoms. Frequency analysis and binary and multinomial regression were employed for data analysis. The prevalence of mild depressive symptoms increased from 23.1% before the pandemic to 35.1% during the pandemic. Our findings reveal that practicing PA before the pandemic was a protective factor (OR: 0.19; 95% CI: 0.13, 0.30; *p* < 0.001) against mild depressive symptoms. Additionally, individuals who continued to practice PA during the pandemic had a lower chance of presenting mild (OR: 0.21; 95% CI: 0.15, 0.30) and moderate/severe (OR: 0.15; 95% CI: 0.08, 0.27) symptoms. Furthermore, our study shows that PA, which was already a protective factor before the pandemic, remained protective during the pandemic, even for those with the highest levels of depression.

## 1. Introduction

Social distancing is one of the key strategies to prevent the spreading of COVID-19, but one possible result is the rise in mental health problems [1]. The lack of social life combined with factors such as the fear of being contaminated, financial instability from economic crises, reduction in exercise, increased sedentary behavior, and the collective mourning for thousands of people who died due to COVID-19 have had a direct impact on the health of the general population [2].

The lockdown imposed during the COVID-19 pandemic has been linked to various adverse health outcomes, including reduced physical activity, weight gain, depression, and cardiovascular drug treatment [2,3]. Notably, mental disorders have been highly prevalent, with depression alone affecting nearly 38% of the population [4]. A small cross-sectional study conducted during the first wave of the pandemic showed a high prevalence of anxiety and depression in young adults, particularly females who had lived in lockdown for over three months [5]. Additionally, it is important to acknowledge that sex and personality are significant factors associated with attitudes toward the pandemic, negative affect, and emotional instability [6].

Research efforts have been directed toward developing strategies to address the challenges posed by the COVID-19 pandemic, such as social isolation and mental disorders. One such strategy is physical activity (PA), which has been investigated in several studies. For example, a cross-sectional study conducted in Greece during the second wave of the pandemic found a significant association between PA, depression, and quality of life [7]. The authors reported negative and positive correlations between PA and depression and PA and quality of life, respectively, highlighting the crucial role of PA in promoting good mental health.

Similarly, a study in France revealed that a large proportion of individuals (55.1% of men and 58% of women) experienced reduced levels of PA during lockdown [8]. However, a small subset of participants reported increased levels, with 19–23% of women showing improvements in depressive symptoms. These findings underscore the potential benefits of maintaining or increasing PA levels, particularly among individuals experiencing mental health challenges during the pandemic.

The adverse mental health effects of pandemics have underscored the need for innovative interventions to mitigate these challenges. PA has emerged as a promising intervention [8,9]. International PA guidelines recommend a minimum of 150 min of moderate or 75 min of vigorous-intensity PA per week for optimal physical and mental health benefits [9]. Previous studies have also shown that regular PA is associated with protective effects against depression [10].

However, evidence from multiple countries suggests that PA levels have declined during the pandemic [8,11,12]. This decline in PA levels is concerning, given the potential impact on mental health outcomes, and highlights the urgent need to develop strategies to encourage individuals to maintain or increase their PA levels during this time.

Several studies have examined the prevalence and incidence of depression and its relationship with PA, with most relying on web-based survey questionnaires [13,14]. While web-based surveys are a valuable research tool, their uncertain measures have raised concerns [15]. Furthermore, studies using alternative instruments or strategies to minimize bias and evaluate mental health in the general population during the COVID-19 pandemic remain limited.

To address this research gap, the present study sought to investigate the impact of the COVID-19 pandemic on depressive symptomatology in the general adult population. Specifically, the study aimed to assess the association between PA and depression using in-person contact and online face-to-face data collection to minimize the potential biases of web-based surveys.

## 2. Materials and Methods

### 2.1. Study Design

The present cross-sectional study consisted of two phases, and was a component of the larger research project entitled “Epidemiological Profile of Obesity and Related Factors in the Southwest Region of Bahia,” conducted in Vitória da Conquista, Bahia, Brazil. The study protocol was reviewed and approved by the Research Ethics Committee of Faculdade Independente do Nordeste (Fainor) under the reference numbers 1,859,545/2016 and 4,737,274/2021. Before their inclusion in the study, all participants provided informed consent by signing the relevant form.

### 2.2. Context

The data were collected at two different moments: Phase (1) between May 2018 and June 2019, before the COVID-19 pandemic, and Phase (2) during the COVID-19 pandemic, between August and September 2020. Participants were interviewed in person in the first data collection phase (before the pandemic). In the second phase of the study (during the pandemic), participants were interviewed via videoconference through Google Meet. We utilized the participants’ previously recorded data from the project above.

### 2.3. Participants

In the initial phase of the study, the sample comprised 2000 individuals from the population of Vitória da Conquista, which has a total of 343,643 inhabitants. Recruitment was carried out through advertisements in public spaces and telephone calls utilizing health service databases. For the second phase, we used data collected in Phase 1 to contact individuals by phone and invite them to participate.

The sample size for this study was calculated using the PPGCS Sample Size calculator [16] considering the total population of the city (343,643 inhabitants), a 99% confidence level, a 5% estimation error, and a 50% estimated prevalence of depression. Based on these parameters, the estimated sample size was 665 individuals. To account for potential dropouts and missing data, we aimed to recruit 20% more participants for a total of 797 individuals to maintain adequate statistical power.

We established specific inclusion and exclusion criteria for this study to select the appropriate data for analysis. The inclusion criteria included i) participants of both genders, ii) no visual or auditory impairments, and iii) preserved physical functions. Individuals under 17 or over 80 were excluded from the study because they fell outside the age range specified for using BDI [17]. Participants who could not be reached via videoconference or failed to respond during the COVID-19 pandemic were also excluded. Ultimately, a total of 785 participants (120 more than the estimated sample) ranging in age from 18 to 76 years (male: 18–76 years; female: 18–71 years) were included in the study. The age cutoff point of 60 years was used to classify elderly individuals [18].

### 2.4. Instruments and Variables

The research staff consisted of four trained psychologists who conducted the interviews and administered the instruments. Before data collection, the psychologists underwent training sessions to ensure consistency and standardization in the application of each instrument. Participants were contacted by phone, and interview appointments were scheduled after the initial contact.

During this study, interviews lasting 40 to 60 min were conducted with participants. Initially, an anamnesis was performed, and data about socio-demographic variables such as age (in years), sex (male or female), marital status (single, married, divorced, or widowed), employment situation (working or not), family income (<1 salary, 1–3 salaries, or >3 salaries) were collected. To evaluate the presence and intensity of depressive symptoms, the Beck Depression Inventory (2nd Edition) was utilized [17]. This self-report scale comprises 21 items, each with four alternatives, reflecting increasing degrees of depression severity. Scores range from 0 to 63, with higher scores indicating greater levels of depression severity. Specifically, scores of 0 to 9 indicate minimal depression, scores of 10 to 18 suggest mild depression, scores of 19 to 29 suggest moderate depression and scores of 30 to 63 suggest severe depression. For this study, the valid (Intraclass Correlation Coefficient = 0.89) and reliable (Cronbach’s alpha coefficient = 0.93) Brazilian version of the BDI-II for community-dwelling adults was employed [19].

### 2.5. Statistical Analysis

This study conducted a descriptive analysis to examine the primary variables. Absolute and relative frequencies that aligned with the research objectives were obtained. To compare participant frequencies by age classification (adult/elderly) and sex (male/female), the chi-square test was applied.

Furthermore, binary logistic regression was conducted for the outcome variable. The moment before the pandemic was considered for this analysis, as it was when individuals with minimal and mild symptoms could be identified. During the pandemic, multinomial logistic regression was applied for the outcome variable. For this analysis, ‘minimal symptoms’ was selected as the reference category, while mild and moderate/severe symptoms were selected as comparison categories. Both multiple analyses were related to physical activity and were adjusted for confounding variables such as sex and age.

All analyses were conducted using the SPSS 22.0 software (IBM Corp.: Armonk, NY, USA), and a significance level of 0.05 and a 95% confidence interval (95% CI) were established.

## 3. Results

In this study, 785 participants with an average age of 37.4 ± 13.2 years were evaluated, with the majority (72.5%) being females. Of these, 23.1% had mild depression before the pandemic, and this percentage increased to 35.1% during the pandemic (see Table 1).

Regarding the levels of depressive symptoms, the results of the binary logistic regression analysis, adjusted for sex and age, showed that engaging in physical activity before the pandemic was associated with a lower chance (OR: 0.19; 95% CI: 0.13, 0.30; *p* < 0.001) of experiencing mild depressive symptoms. Similarly, the analysis adjusted for sex and age during the pandemic revealed that individuals who were practicing physical activity had a lower chance of presenting with mild (OR: 0.21; 95% CI: 0.15, 0.30) and moderate/severe (OR: 0.15; 95% CI: 0.08, 0.27) depressive symptoms compared to those who were not (see Table 2).

## 4. Discussion

The present study aimed to investigate the impact of the COVID-19 pandemic on depression and the role of physical activity (PA) in Brazilian individuals. A total of 785 participants aged 37.4 ± 13.2 years were evaluated, most of which (72.5%) were females. The results showed that depression worsened during the pandemic, with an increase in the percentage of mild (35.1%) and moderate/severe (10.6%) symptoms. However, individuals practicing PA before or during the pandemic had lower chances of presenting depressive symptoms. These findings are consistent with previous studies showing the association between a sedentary lifestyle, mental health disorders, and low levels of PA [20,21,22]. This study also highlights the importance of maintaining an active lifestyle during the pandemic to mitigate the negative impacts on mental health. However, further large clinical trials are needed to confirm the hypothesis that PA is a protective factor against depression during the COVID-19 pandemic.

The present study found no significant interaction effects between sex or age and the association between PA and moderate/severe depressive symptoms. However, the results did reveal that women had a significantly higher likelihood of experiencing mild depressive symptoms than men, with a two-fold increased risk. This finding is consistent with previous research, highlighting that women and young adults are at a greater risk of developing mental health disorders during social isolation [23]. Notably, physically active women appear to have lower depressive symptom scores, as assessed by the BDI-II, even during the COVID-19 pandemic [19]. Our findings suggest that PA may represent a protective factor for mental health, irrespective of age and sex.

Several clinical studies have provided evidence of the antidepressant effect of physical activity (PA) [24]. Although the neurobiological mechanisms underlying this effect are diverse and not yet fully understood, it is known that exercise stimulates muscles to synthesize various substances, including Brain-Derived Neurotrophic Factor (BDNF), vascular endothelial growth factor (VEGF), anti-inflammatory cytokines, and myokines that promote neuronal survival and the production of neurotransmitters. These neurotransmitters, including serotonin, norepinephrine, and dopamine, are essential mood regulators in the cortex and limbic system [25]. However, it should be noted that the present study did not investigate these mechanisms, and therefore, caution is advised when interpreting these findings. Nevertheless, the associations identified in the present study have significant implications for developing and promoting public policies that encourage physical activity to improve overall health [26].

## 5. Limitations and Future Directions

The present study, while yielding encouraging results, has limitations. Firstly, it is important to acknowledge that the measurement of depressive symptoms via the BDI does not necessarily indicate a diagnosis of a Mood Disorder. Accurate diagnoses require psychiatric clinical criteria, individual classification, and the application of different psychopathological protocols [27]. Additionally, our study lacks an instrument for assessing the participants’ physical activity levels. However, it is important to note that due to the chaotic period in which the research was conducted and the fact that interviews were conducted via videoconference, there were limitations on the inclusion of instruments of this nature. This new scenario must have allowed for the adequate evaluation of the mental burden of conducting a complex interview remotely. Therefore, we chose to minimize the participant burden as much as possible.

Acknowledging the challenges associated with conducting clinical trials with interventions during pandemic periods is essential. Therefore, online face-to-face interviews are considered one of the most effective study designs to minimize bias. For future research, we recommend including a web survey to measure physical activity levels and other lifestyle factors such as sleep and diet. Further studies that reinforce these findings could assist health professionals and policymakers in developing future strategies to address pandemic-related issues.

## 6. Conclusions

The COVID-19 pandemic has exacerbated depressive symptoms among adults, resulting in an increased prevalence of depression. However, physical activity (PA) appears to have served as a protective factor against depression both before and during the pandemic, even in cases of severe depression. Additional research is required to explore the potential of an active lifestyle as a low-cost intervention that could be implemented as a public policy to mitigate the adverse impact of pandemic-related mental disorders in the future.

## Figures and Tables

**Table 1:** Descriptive analysis of the study participants. (*n* = 785).

Variables	*n*	%	*p*-Value ^#^
Sex			
Male	216	27.5	<0.001
Female	569	72.5	
Age classification			
Adult	724	92.2	<0.001
Elderly	61	7.8	
Marital status *			
Single	415	53	
Married	305	39	
Divorced	51	6.5	
Widow(er)	12	1.5	
Depressive symptoms before the pandemic *			
Minimal	599	76.9	
Mild	180	23.1	
Depressive symptoms after the pandemic *			
Minimal	426	54.3	
Mild	275	35.1	
Moderate/Severe	83	10.6	

* Variation in *n* due to loss of information. ^#^ Chi-square.

**Table 2:** Multinomial Logistic Regression. Depressive symptoms (DS) during the pandemic, with participants with minimal symptoms used as the reference category.

**Initial Model**	**Mild DS**		**Moderate/Severe DS**	
**Variables**	**OR (CI_95%_)**		**OR (CI_95%_)**	
PA _during the pandemic_		<0.001		<0.001
No	1.00		1.00	
Yes	0.21 (0.15, 0.29)		0.15 (0.08, 0.27)	
Final Model	Mild DS		Moderate/Severe DS	
Variables	OR _adjusted_ (CI_95%_) ^#^		OR _adjusted_ (CI_95%_) ^#^	
PA _during the pandemic_		<0.001		<0.001
No	1.00		1.00	
Yes	0.21 (0.15, 0.30)		0.15 (0.08, 0.27)	
Sex				
Male	1.00		1.00	
Female	2.00 (1.36, 2.94)	<0.001	1.61 (0.91, 2.85)	0.102
Age _classification_				
Elderly	1.00		1.00	
Adult	0.91 (0.51, 1.63)	0.754	2.54 (0.74, 8.73)	1.38

^#^ The analysis of PA and DS was adjusted for sex and age. Goodness-of-fit test *p*-value = 0.106.

## Data Availability

The data presented in this study are available from the corresponding author upon reasonable request.
